# Denials, Surprise Charges, Starting Over: How Medicare Recipients Navigate the Medicare Mental Health Coverage Gap

**DOI:** 10.1093/geroni/igaa057.309

**Published:** 2020-12-16

**Authors:** Matthew Fullen, Megan Dolbin-MacNab, Nancy Brossoie, Jonathan Wiley, Gerard Lawson

**Affiliations:** 1 Virginia Tech, Blacksburg, Virginia, United States; 2 Marietta College, Radford, Virginia, United States

## Abstract

Medicare is the primary insurance provider for approximately 51 million older adults, including those who seek mental health care. Medicare provider eligibility was last updated in 1989, and approximately one-third of the graduate-level mental health workforce (i.e., Licensed Professional Counselors and Licensed Marriage and Family Therapists) is excluded from Medicare, despite these professionals participating in Medicaid, TRICARE, the Veterans Administration, and private insurance plans. This Medicare mental health coverage gap (MMHCG) raises concerns about older adults’ access to mental health care, resulting in a policy misalignment between Medicare’s provider regulations and a growing number of older adults seeking mental health care. However, little is known about the precise impact of the MMHCG. To better understand how the MMHCG impacts older adults, we interviewed 17 Medicare-insured individuals about their experiences accessing mental health services. Using a phenomenological framework to analyze our data, we found that Medicare recipients described several consequences, such as: 1) a detrimental impact on their mental health and well-being; 2) concerns about having to start over with new providers due to commencing mental health treatment only to have services interrupted once the provider is no longer Medicare-reimbursable; and 3) relying on pro bono services from Medicare-excluded providers with uncertainty about the long-term sustainability of these arrangements. The presenters will describe how these findings fit within the current Medicare mental health service context, including the direct impact on older adults’ mental health. Discussion will also focus on policy implications of the findings and possible solutions for addressing the MMHCG.

